# Understanding the Degradation of Core-Shell Nanogels Using Asymmetrical Flow Field Flow Fractionation

**DOI:** 10.3390/jfb14070346

**Published:** 2023-06-29

**Authors:** Edyta Niezabitowska, Dominic M. Gray, Eduardo Gallardo-Toledo, Andrew Owen, Steve P. Rannard, Tom O. McDonald

**Affiliations:** 1 Department of Chemistry, University of Liverpool, Crown Street, Liverpool L69 7ZD, UK; 2 Department of Pharmacology and Therapeutics, Institute of Systems, Molecular and Integrative Biology, University of Liverpool, Liverpool L3 5TR, UK; 3 Centre of Excellence in Long-Acting Therapeutics (CELT), University of Liverpool, Liverpool L3 5TR, UK; 4 Materials Innovation Factory, University of Liverpool, Liverpool L7 3NY, UK; 5 Department of Materials, The University of Manchester, Oxford Road, Manchester M13 9PL, UK; 6 Henry Royce Institute, The University of Manchester, Oxford Road, Manchester M13 9PL, UK

**Keywords:** asymmetrical flow field flow fractionation, nanogel, degradation, core-shell, poly(N-isopropylmethacrylamide), thermoresponsive

## Abstract

Nanogels are candidates for biomedical applications, and core-shell nanogels offer the potential to tune thermoresponsive behaviour with the capacity for extensive degradation. These properties were achieved by the combination of a core of poly(N-isopropylmethacrylamide) and a shell of poly(N-isopropylacrylamide), both crosslinked with the degradable crosslinker N,N′-bis(acryloyl)cystamine. In this work, the degradation behaviour of these nanogels was characterised using asymmetric flow field flow fractionation coupled with multi-angle and dynamic light scattering. By monitoring the degradation products of the nanogels in real-time, it was possible to identify three distinct stages of degradation: nanogel swelling, nanogel fragmentation, and nanogel fragment degradation. The results indicate that the core-shell nanogels degrade slower than their non-core-shell counterparts, possibly due to a higher degree of self-crosslinking reactions occurring in the shell. The majority of the degradation products had molecule weights below 10 kDa, which suggests that they may be cleared through the kidneys. This study provides important insights into the design and characterisation of degradable nanogels for biomedical applications, highlighting the need for accurate characterisation techniques to measure the potential biological impact of nanogel degradation products.

## 1. Introduction

Nanogels, sometimes also referred to as microgels, are submicron three-dimensional hydrogel particles, comprising of a crosslinked polymer network that swells in a good solvent [1]. Many nanogels can exhibit stimuli responsive behaviour and poly(N-isopropylacrylamide) (PNIPAM) nanogels are one of the most widely studied thermoresponsive nanogels [2,3,4]. This interest in PNIPAM nanogels is due to their volume phase transition temperature (VPTT) of 32 °C, which is close to body temperature [5]. The responsive behaviour of nanogels can be used to provide useful properties, such as triggered release [6], and to activate particle aggregation behaviour [7]. As a result, there has been considerable interest in using PNIPAM nanogels for biomedical applications such as in-situ-forming implants [8,9,10] and drug delivery systems [8,11]. However, a challenge for PNIPAM nanogels for in vivo applications is their non-degradable nature, necessitating surgical removal with associated complexity and cost. One approach to provide degradability is to use N,N′-bis(acryloyl)cystamine (BAC) as the crosslinker. This crosslinker introduces disulphide bonds between polymer chains which can be broken in the presence of reductant dithiothreitol (DTT), a molecule that has been used to mimic the role of glutathione (GSH) in the body [12]. Upon the cleavage of the crosslinkers, the degradation of nanogels occurs, resulting in the generation of low molecular weight polymers which should be cleared from the body. Additionally, BAC-crosslinked nanogels have been shown to present no cytotoxicity at concentrations of up to 3 µM, matching the behavior of nanogels produced with no-degradable crosslinkers [13]. However, obtaining PNIPAM nanogels that show complete degradation has been challenging as PNIPAM nanogels often form permanent crosslinks due to a hypothesised chain transfer reaction on the polymer backbone [14]. This chain transfer can be greatly reduced by the use of a methacrylamide such as N-isopropylmethacrylamide (NIPMAM) [15,16]. However, the resulting PNIPMAM nanogels have a VPTT of ~43 °C which can make the nanogels less suited to biomedical applications. It has previously been shown that core-shell nanogels can be produced by the controlled addition of different monomers/crosslinkers [17,18]. Based on these approaches, we have shown that, by preparing core-shell nanogels with PNIPMAM in the core and PNIPAM in the shell, it is possible to tune the VPTT while also achieving near complete degradation [19]. The core-shell structure of these nanogels means that the degradation behaviour may differ from nanogels with a single composition.

It is important to be able to understand the degradation behaviour of nanogels, as changes in size during the degradation process would have the potential to change their biological impact and/or pharmaceutical behaviour. A considerable challenge in characterising the degradation behaviour of nanoparticles is the ability to measure the change in particle sizes whilst simultaneously detecting the formation of any water-soluble products formed as the particles degrade/dissolve. Typically, degradation behaviour is measured in the bulk dispersion using light scattering techniques such as dynamic light scattering (DLS) [19,20,21,22] or multiangle light scattering (MALS) [12,23,24]. However, due to the intensity of scattered light being approximately proportional to the sixth power of the radius, the light scattering from the larger nanoparticles in a distribution can be too intense to allow the detection of the much smaller nanogel fragments or soluble polymers [25]. As such, observing the nature of degradation in microgels is difficult using techniques that average across the bulk dispersed sample. Indeed, it was not possible to resolve the smaller particulates formed during the degradation process by DLS analysis of the bulk sample [19]. One approach to address this limitation has been shown by South and Lyon, who used atomic force microscopy to make direct observations on particle changes during degradation [26]. They showed that the nanogels would initially show a slight swelling response before reducing in size as the polymer is lost from the particles. Unfortunately, this method is only suited to nanogels that are adsorbed onto a surface and cannot be used to characterise dispersed materials.

One technique that allows the accurate, high-resolution characterisation of polydisperse colloidal samples is asymmetric flow field flow fractionation (AF4). This technique allows the separation of particles and molecules based on their diffusion coefficients. AF4 separation and analysis has been gaining interest in the field of nanomedicine and nanoscience [27,28,29], with increasing reports of separation nanogels and polymers by AF4 [30,31,32]. Using AF4 and measuring both the radius of gyration (R_g_) (using MALS) and the hydrodynamic radius (R_h_) (using DLS), differences in the internal crosslinking densities of differently sized nanogels could be determined [33].

The ratio of Rg/Rh can provide insights into the internal structure and shape of particles [33,34,35]. Surprisingly, there are few papers that have studied the degradation of nanogels by AF4 [12,16]. Smith et al. [16] conducted a study where they synthesised PNIPAM nanogels with a degradable crosslinker called N,O-dimethacryloylhydroxylamine. The nanogels degradation process was examined under various pH and temperature conditions, and subsequently characterised using AF4-MALS-DLS measurements over time. The resulting data revealed a reduction in the normalised scattering intensity in AF4-MALS measurements of nanogels incubated at 37 °C and pH values above 7, indicating a faster degradation rate at higher pH values. MALS90° signal measurements performed before and after degradation exhibited changes in intensity, thus qualitatively demonstrating the extent of nanogel degradation. In a subsequent study, Gaulding et al. [12] demonstrated the synthesis of PNIPAM nanogels using a disulphide-based crosslinker BAC through a redox-initiated precipitation polymerisation method. AF4-MALS and batch DLS measurements were employed to characterise the nanogels. The degradation of particles was induced by DTT and evaluated using AF4-MALS in a phosphate buffer. A decrease in the light scattering intensity and a reduction in the molecular mass of the nanogels was observed, showing that nanogel swelling and mass loss were occurring. Therefore, AF4 measurements demonstrated potential in analysing the degradation of PNIPAM nanogels and proved to be more effective than batch DLS analysis due to the fractionation of particles allowing an analysis of all the particle size ranges within the samples. Additionally, it is attractive to use AF4-MALS-DLS to analyse a nanogel sample during the degradation process so as to characterise the temporal changes in the structure of the particles as they degrade.

**Figure 1:**
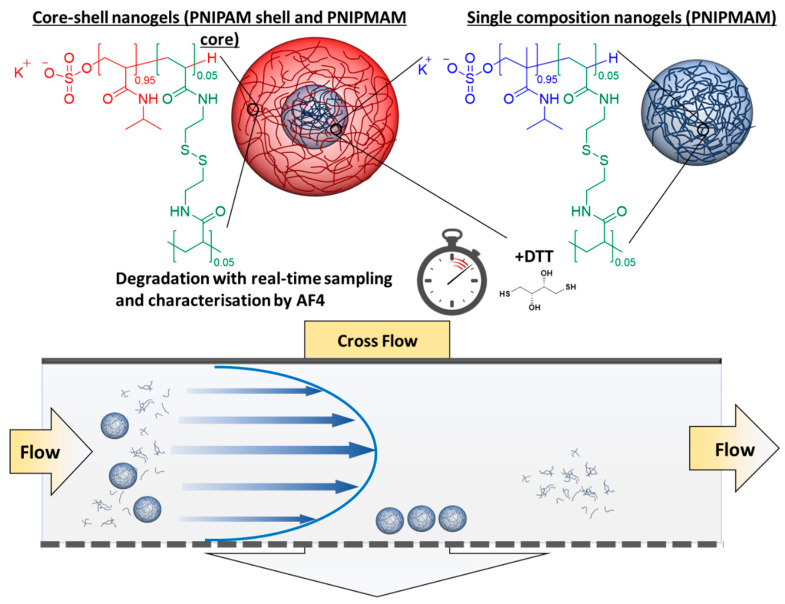
Monitoring the real-time degradation behaviour of core-shell and single composition nanogels in the presence of DTT by using AF4. Core-shell nanogels were synthesised with a core of PNIPMAM (blue) and a shell of PNIPAM (red), the single composition nanogels were only made of PNIPMAM. Both PNIPMAM and PNIPAM were crosslinked with the same BAC crosslinker (green) in the same ratio to the monomers.

In this work, the degradation behaviour and mechanism of core-shell nanogels, comprising PNIPAM and PNIPMAM, was studied through the use of AF4-MALS-DLS (Figure 1). Dispersion polymerisation with BAC as the crosslinker was used to produce the nanogels. Two different nanogel compositions were analysed; one sample was a single composition nanogel made of PNIPMAM and the crosslinker, while the second sample was a core-shell nanogel made of a shell of PNIPAM and a core of PNIPMAM. The degradation of the nanogels was triggered with DTT and the samples were analysed using AF4 throughout the different stages of degradation. This approach provided a unique insight into how the composition of the core-shell nanogels influenced their real-time degradation, which is important for the future rational design of pharmaceutical products which control xenobiotic release.

## 2. Experimental

### 2.1. Materials

Sigma–Aldrich Company Ltd., Gillingham (Dorset) UK, a subsidiary of Merck KGaA, Darmstadt, Germany, provided the following chemicals: potassium persulfate (KPS, ≥99%), anhydrous sodium hydroxide pellets (NaOH, analysis grade), sodium dodecyl sulphate (SDS, ≥99%), N-Isopropylacrylamide (NIPAM, ≥99%), N-isopropylmethacrylamide (NIPMAM, 97%), N,N′-bis(acryloyl)cystamine (BAC, 98%), 1,4-dithiothreitol (DTT, >97%), and deuterium oxide (99.9% atom D, containing 1 wt% 3-(trimethylsilyl)-1-propanesulfonic acid, sodium salt). All chemicals were used as received. Additionally, Type I distilled water, with a resistivity of >18 MΩ cm^−1^ (PURELAB option R, Veolia), was used. Spectrum Europe B.V., Breda, the Netherlands, supplied the Spectra/por 2 dialysis tubing with a molecular weight cutoff (MWCO) of 12–14 kDa. A Corning bottle top vacuum filter system with cellulose acetate membrane (pore size 0.22 µm) was obtained from Sigma–Aldrich Company Ltd., Gillingham (Dorset) UK, a subsidiary of Merck KGaA, Darmstadt, Germany. Finally, NovaChem was purchased from Postnova Analytics Ltd.

### 2.2. Nanogel Synthesis

Dispersion polymerization was used to produce the nanogels. Table 1 presents the composition used in the synthesis of nanogels. The BAC cross-linker, SDS surfactant, and monomer (NIPAM or NIPMAM) were dissolved in distilled water in a 250 mL two-neck round bottom flask with reflux condenser. The solution was degassed by bubbling with nitrogen for 1 h whilst magnetically stirring at 400 rpm. Separately, KPS initiator was dissolved in distilled water and purged with nitrogen for 1 h. The solution containing the monomers, BAC and SDS, was then heated to 70 °C, and the solution of KPS was added to initiate the polymerisation. The reaction proceeded under a blanket of nitrogen gas for 1 h at 70 °C, after which the shell monomer solution was added along with further KPS initiator solution. The monomer solution contained the NIPAM along with BAC and SDS. The monomer solution and initiator solution were separately degassed with nitrogen for 1 h whilst stirring (400 rpm). After a further 3 h of polymerisation at 70 °C, the heating was turned off and solution was cooled down to room temperature. The nanogel suspension was purified by dialysis for 5 days using regenerated cellulose dialysis tubing (12–14 kDa MWCO, Spectrum Labs, Fisher Scientific, UK), the distilled water was replaced twice daily in order to remove unreacted monomers and residue surfactant. After dialysis, samples were dried by lyophilised using a Virtis benchtop K for 72 h. The samples were subsequently redispersed with shaking at the required concentrations.

### 2.3. Separation Systems and Conditions

The experimental setup for AF4 analysis involved employing an AF2000MT instrument with RI and UV-Vis detectors from Postnova Analytics, based in Landsberg, Germany. A MALS detector (PN3621, Postnova) with 21 angles (ranging from 7° to 164°) and operating at a laser wavelength of 532 nm was connected in-line with AF4. The system utilised an autosampler (PN5300, Postnova). The R_h_ of the samples were determined using DLS with a Malvern Zetasizer Nano ZS instrument (running Malvern Zetasizer software V7.12) from Malvern Instruments, located in Malvern, UK. The instrument employed a 633 nm He–Ne laser, and the detector was positioned at an angle of 173°. DLS measurements were conducted using the Malvern quartz flow cell (ZEN0023) at a flow rate of 0.5 mL min-1 and a temperature of 28 °C, which was connected in-line with the AF2000MT. The AF4 separation channel consisted of a 350 µm spacer and a 10 kDa regenerated cellulose membrane. The eluent used was composed of 0.2% NovaChem in Milli-Q H_2_O. Type I distilled water with a resistivity greater than 18 MΩ cm^−1^ (PURELAB option R, Veolia) was obtained from a water purification system. The eluents were filtered using a Corning bottle top vacuum filter system equipped with a cellulose acetate membrane featuring a pore size of 0.22 µm. The sample injection volume was 5 µL of a 1 mg mL^−1^ solution, performed by the autosampler. The UV-Vis detector measured wavelengths of 250 nm and 300 nm. The separation conditions were as follows: the injection/focusing time was set to 3 min with a range of cross-flows from 2 to 0.1 mL min^−1^. The chosen cross-flow rate remained constant for the initial 0.2 min (t_0_–t_0.2_) and, subsequently, the cross-flow decreased exponentially (exponent 0.2) from its initial value to 0.1 over a period of 40 min. After the complete reduction in cross-flow, the tip-flow of 0.1 mL min^−1^ continued for an additional 20 min. Throughout the injection, focusing, and separation steps, a constant detector flow rate of 0.5 mL min^−1^ was maintained. The data processing was carried out using AF2000 software (Version 2.1.0.5). The R_g_ values were fitted to random coil model which gave the best fit. The size distributions of the samples were determined from the R_g_ data using an average 20 and points 200.

### 2.4. Dynamic Light Scattering (DLS)

To characterise the nanogels post-synthesis, DLS measurements were conducted at 25 °C using a 1 mg mL^−1^ nanogel dispersion. The measurements were performed with a Malvern Zetasizer Nano ZS instrument (running Malvern Zetasizer software V7.12) from Malvern Instruments, located in Malvern, UK. The instrument used a 633 nm He–Ne laser with the detector positioned at 173°. The equilibration time was set to 240 s, unless otherwise specified. The refractive index of the material was set to 1.520. The measurement parameters, such as run number, duration, measurement position, and attenuator selection, were automatically chosen. Disposable polystyrene cuvettes with a path length of 1 cm were employed for the measurements. The measurements were performed in triplicate, and the Z-average diameter and polydispersity index (PDI) values were obtained through cumulants analysis, while the size distributions were derived from the distribution fit using the general-purpose analysis model.

### 2.5. Degradation of Nanogels

Samples were analysed at 1 mg mL^−1^ in phosphate buffered saline (pH 7). Degradation was achieved using 10 mM DTT concentration to degrade particles. The samples were placed in autosampler (HPLC) vials at ambient temperature, these vials were sampled at set time intervals using the AF4 autosampler.

## 3. Results and Discussion

Two nanogel compositions were synthesised by free radical dispersion polymerisation; one with a single composition containing PNIPMAM and the BAC crosslinker (referred to as single composition nanogels from here on), while the second sample was a core-shell nanogel comprising a core of PNIPMAM and a shell of PNIPAM (from here on referred to as core-shell nanogels). The core-shell nanogels had a molar composition of 15:85 PNIPMAM:PNIPAM and the BAC crosslinker was used at the same concentration (5 mol% of the total monomers) in both the core and the shell. This composition was selected based on prior work showing that samples of this type displayed a high degradability based on a batch DLS measurement using residual derived count rates (suggesting 97.5% degradation) [19].

### 3.1. Characterisation of the Single Composition and Core-Shell Nanogel Samples

**Figure 2:**
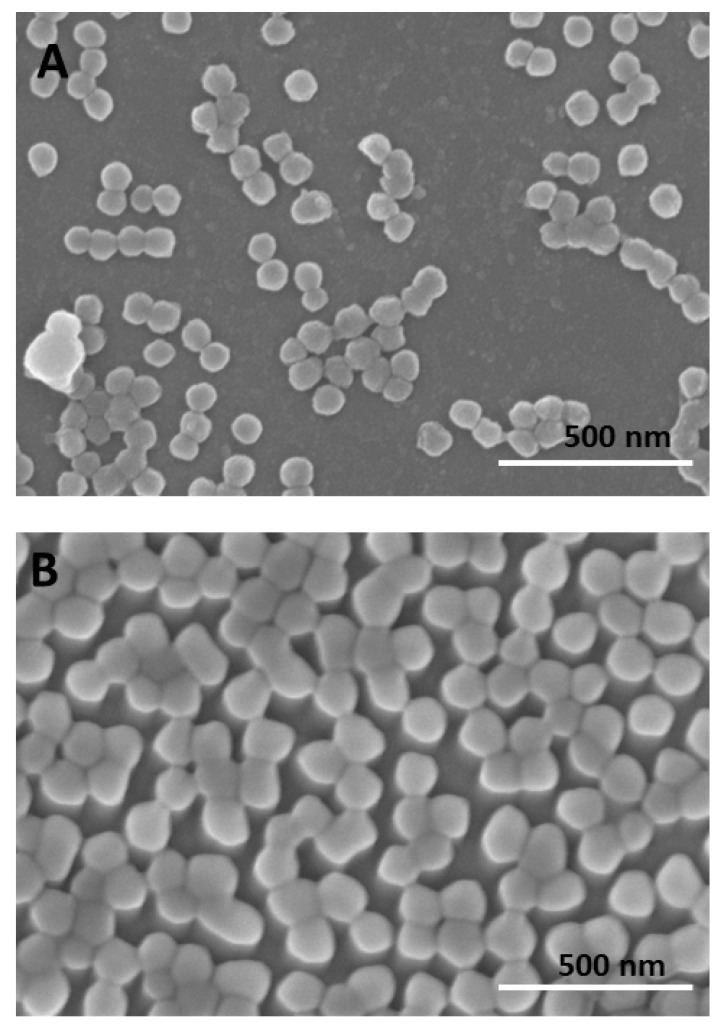
SEM analysis of the two nanogel samples. (**A**) SEM images for the core-shell nanogels. (**B**) SEM images for single composition nanogels.

Both the single composition and core-shell nanogel samples were initially characterised by batch DLS and were found to be monomodal with a narrow dispersity; a mean radius of 94 ± 1 nm and polydispersity indexes (PDI) 0.11 ± 0.01 was measured for the core-shell nanogel, and a 72 ± 2 nm mean radius and PDI 0.02 ± 0.02 was measured for the single composition nanogel (ESI Figure S1 shows the particle size distributions). Scanning electron microscopy (SEM) analysis of the dried samples showed uniform particles with an approximate 40 nm radius for the core-shell nanogel and a 58 nm radius for the single composition nanogel (Figure 2). The core-shell nanogel sample also displayed objects with radii smaller than ~10 nm; however, these could not be individually resolved.

**Figure 3:**
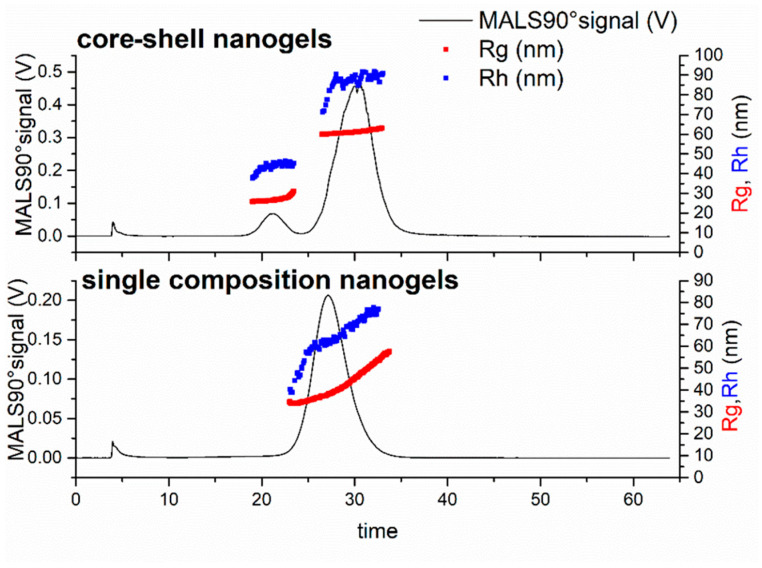
AF4-MALS-DLS fractograms for core-shell nanogels and the single composition nanogels before degradation. The data present the MALS90° signal, R_g_ and R_h_ for each of the samples.

The two nanogels samples were also characterised by AF4 coupled to MALS and DLS detectors (AF4-MALS-DLS), using a method previously shown in the literature [29]. The eluent 0.2% NovaChem was used to fractionate nanogels and their degradation products and reproducibility was assessed using three injections of each of sample. Both nanogels displayed high reproducibility with closely overlaying fractograms (ESI Figure S2). The fractograms of each of the samples (Figure 3) shows elution time versus MALS90° signal and R_g_, R_h_.

For AF4, particles with larger hydrodynamic sizes elute later due to the separation behaviour in the channel. A void peak was seen at an elution time of ~4 min corresponding to the end of the focussing stage of the method. The elution for core-shell nanogels showed a bimodal distribution within the AF4 measurements. The elution of the first peak started at 18 min and finished at 22 min, followed by a second peak at 25 min and finishing at 35 min. This analysis showed the benefit of the AF4 analysis compared to batch DLS alone; the core-shell nanogel sample had appeared monomodal by batch DLS analysis. The bimodal nature of the core-shell nanogels may be a result of the PNIPMAM core not uniformly adding the outer PNIPAM layer during the shell polymerisation step. The single composition nanogel sample was found to be monomodal with elution starting at 22 min and finishing at 32 min. The mode values of R_g_ and R_h_ obtained for both samples from the AF4 measurements are presented in Table 2. The mode values for R_h_ measured on the AF4 were slightly smaller (~10 nm) than those measured on batch DLS (see Table S1 for a comparison of radii measurement by the different techniques). This difference between the fractionated sample and the batch DLS measurement may be due to the sensitivity of DLS to larger objects (scattering intensity is proportionate to r^6^) [36]. Therefore, any larger particles in the distribution will have a disproportionate effect on the mean diameter. Additional insight into the structure of the sample populations can be obtained from the R_g_/R_h_ ratio, sometimes known as the shape factor; the literature has reported a value of 0.775 for homogeneous spheres with uniform density [37,38,39], while lower values indicate nanoparticles with a greater density in the core compared to the shell [40]. Previous studies on nanogels have provided a range of R_g_/R_h_ values of 0.5–0.75 [19,24,41]. While PNIPAM-based nanogels, produced by dispersion polymerisation and with a radii of ~50 nm, have been shown to have R_g_/R_h_ values of ~0.6 [19]. The shape factor calculated for the core-shell nanogels gave two different values for the two populations of nanogels. The AF4-MALS-DLS measurements showed that, at the first peak, these core-shell nanogels had lower shape factor (0.61) compared with the species within the second distribution (0.71), indicating a denser core within the smaller population of particles (Table 2) [33].

The core-shell nanogel (PNIPMAM) appeared to possess a consistent density throughout both the core and the shell, likely due to consistent crosslinking density, as the values for shape factor are close to that of a uniform sphere (0.775). The single composition nanogels had a shape factor of 0.72 (Table 2), which compared very favourably with the main peak within the core-shell nanogel sample. Prior work shows that the core-shell nanogels possess a shell layer of PNIPAM as these nanogels possessed an aggregation temperature of 32 °C, the same as that of single composition nanogels based on PNIPAM [19]. The smaller population of particles in the core-shell nanogel sample had a lower shape factor value, which would suggest a more heterogeneous density with a greater density in the core of the particles.

### 3.2. Characterisation of the Degradation of the Nanogels by AF4

The degradation of both nanogel samples was analysed by AF4-MALS-DLS immediately after exposure to a 10 mM solution of DTT in water to give a real-time analysis of degradation behaviour. Nanogels prepared using a non-degradable crosslinker (such as N,N′-methylenebis(acrylamide)) have previously been shown to be stable against DTT degradation and so have not been included in our study of degradation behaviour [18].

#### 3.2.1. Degradation of Single Composition Nanogels

**Figure 4:**
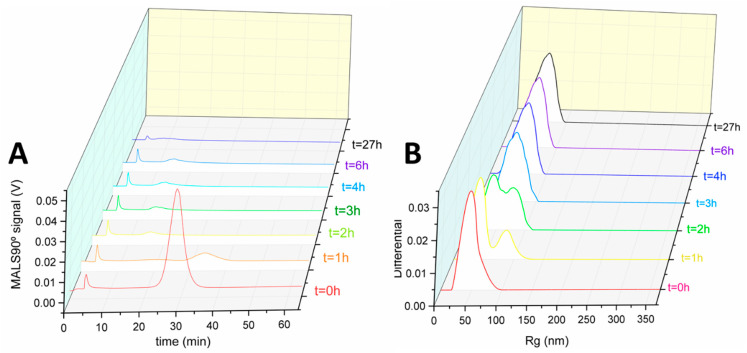
AF4-MALS degradation analysis of the single composition nanogels. (**A**) Fractograms showing the light 90° scattering detector signal at different degradation durations. (**B**) Rg size distribution graphs of the nanogels as determined by MALS at the different sampling timepoints.

For the single composition nanogel sample, the MALS90° signal showed a rapid degradation with continuous changes over the 27 h timeframe of the experiment (Figure 4A); individual fractograms with R_g_ analysis are shown in ESI (Figure S3). The MALS90° signal intensity from the initial monomodal distribution showed an approximate 10-fold decrease in the first hour of degradation and shifted to a later elution time of 32 min. The increase in elution time of the largest peak on the MALS90° signal indicated that nanogels had swelled. This change in size was interpreted as the DTT degrading the crosslinks in the nanogels, resulting in a reduced crosslinking density. The size distributions at each time point are shown in Figure 4B and the population of particles exhibited a mode radius of ~80 nm. This initial swelling behaviour has been reported in other studies on nanogel degradation [16]. The reduction in intensity of the MALS90° signal likely signifies two simultaneous changes in the nanogels: firstly, the swelling and resulting reduction in polymer density in some of the particles reduced the refractive index difference between the nanogels and the surrounding solvent which, in turn, resulted in a reduced light scattering intensity [42]. Secondly, some nanogel particles were breaking up into smaller polymer fragments. This latter explanation is supported by the presence of a low intensity broad peak detected at 20 min (Figure 4A); these particles had a mode radius of ~40 nm (Figure 4B). Interestingly, this R_g_ mode value is almost the same as the mode R_g_ for the nanogel before degradation, however, these objects are unlikely to be undegraded nanogels; the elution time for these objects has reduced by ~8 min, which shows that the particles have a substantially reduced hydrodynamic size. Unfortunately, it was not possible to directly measure the R_h_ values for the smaller population as the scattering in the DLS instrument was too weak. Nonetheless, the smaller population would be expected to possess an increased R_g_/R_h_ value, which may indicate a loosely crosslinked branched structure [43]. The literature has reported R_g_/R_h_ values for non-fractionation samples of branched polymers in the range of 1.1–1.7 [44,45,46]. As the degradation time increased to 2 h, the large population particles were no longer detected and the only peak present was observed at an elution time of 16 min. This peak had a slightly higher intensity than the smaller population that was seen after 1 h of degradation, suggesting an increase in the concentration of objects with a size smaller than the initial nanogels. The reduction in elution time shows that the hydrodynamic size of these objects had further reduced, although the R_g_ size distribution showed a mode of 45 nm (Figure 4B). Ultimately, soluble polymer fragments will be released from nanogels, although an increase in the void peak at 4 min was not observed. Therefore, one could hypothesise that much of the soluble degradation products of these nanogels were below 10 kDa as they were able to pass through the AF4 membrane, causing a decrease in the MALS90° signal. There was little change in the sample between the degradation time of 2 h and 4 h, although the R_g_ size distribution for the sample shifted towards larger values and to a mode of 58 nm. After 27 h, the elution time had further decreased, showing that the particles had further degraded, likely into smaller polymer fragments, as signified by the peak eluting earlier in the analysis run, thus lower R_h_. The R_g_ value after 27 h of degradation was 60 nm. The reduction in the R_h_, while also observing an increase in the R_g_, is likely due to the reduction in the crosslinking of the polymer fragments.

##### Comparison of Single Composition Nanogel Degradation Products with Polymer Prepared in the Absence of Crosslinker

**Figure 5:**
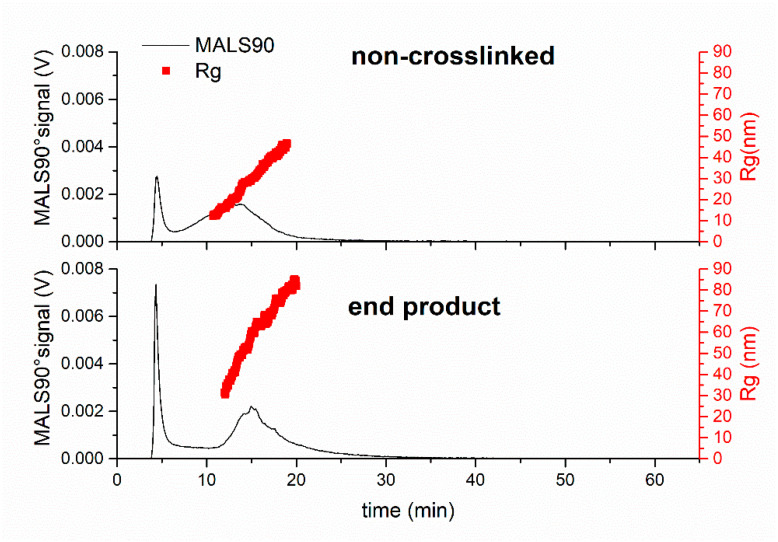
Comparison of the product of degradation of the single composition nanogel after 27 h to a non-crosslinked polymerisation after synthesis, showing that the nanogel was able to degrade to form a product similar to that obtained when no crosslinker was used during polymerisation.

In order to determine if all the degradable crosslinks due to the BAC crosslinker had been degraded, a sample was polymerised without any crosslinker (referred to as non-crosslinked) and also analysed by AF4 and MALS. Unfortunately, R_h_ data could not be obtained as the flow DLS had insufficient sensitivity to measure weakly scattering polymers. The R_g_ values for the end products of single composition nanogels (after 27 h) were also compared with the non-crosslinked PNIPMAM polymer (see Figure 5). Both samples contained polymer that was above 10 kDa (the molecular weight cut-off of the membrane used in the AF4 channel). This can be explained by the self-crosslinking reactions occurring in the PNIPMAM polymerisation. Other studies have shown that that PNIPMAM exhibits less self-crosslinking compared to PNIPAM; nonetheless, PNIPMAM-containing particles can still form intrinsic crosslinks that cannot be degraded using DTT [16]. The products of the degraded single composition nanogel sample were larger than the non-crosslinked sample (mode R_g_ values of 52 nm and 24 nm, respectively). These data suggest that an extensive degradation of the disulphide bonds of the BAC crosslinks in the nanogel has occurred; the polymer that remained was predominately due to permanent self-crosslinking reactions from the polymerisation.

#### 3.2.2. Degradation of Core-Shell Nanogels

**Figure 6:**
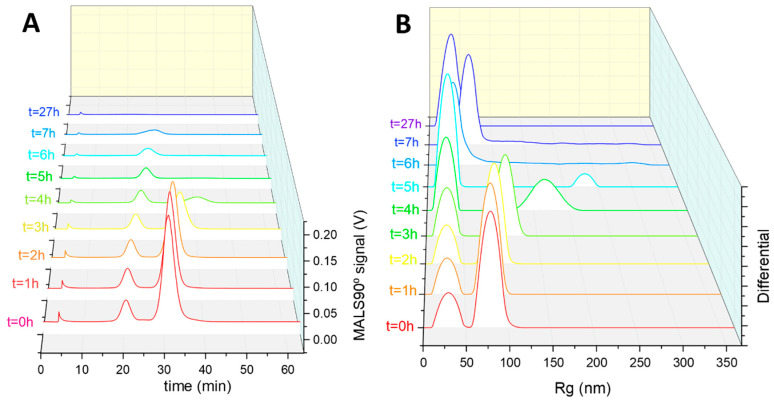
AF4-MALS degradation analysis of the core-shell nanogels. (**A**) Fractograms showing the MALS90° scattering detector signal at different degradation durations. (**B**) R_g_ size distribution graphs of the nanogels, as determined by MALS, at the different sampling timepoints.

The core-shell nanogel sample was also degraded with DTT and monitored by AF4 over 27 h. A comparison of fractograms obtained from the MALS90° signal for the degradation data is shown in Figure 6A (individual fractograms in Figure S4). The core-shell nanogel sample started with its characteristic bimodal distribution, with both peaks shifting slightly to later elution times after 2 h of degradation. As with the single composition nanogel sample, this shift in elution time was due to a simultaneous increase in particle size R_h_ and R_g_ (Figure 6B), which is attributed to the swelling of the nanogels as DTT begins to degrade the crosslinks [19]. A comparison of the R_g_/R_h_ data in the first 2 h of degradation showed limited changes in the values (Figure S5). From 3 h of degradation, the intensity of the second elution peak started decreasing and disappeared completely after 5 h, until the final measurement at 27 h where the smaller nanogel population (first elution peak) continued to show a swelling behaviour (increasing elution time), and then dissolution (as indicated by the disappearance of the peak). This AF4 analysis showed that the process of degradation was quicker for the second nanogel population compared with material comprising the first peak, presumably due to a different internal structure or internal density. As seen from the shape factor analysis, the smaller population of nanogels possessed a core-shell type structure, potentially indicating that a more densely crosslinked core delays the action of DTT and impacts the cleavage of disulphide bonds in BAC units.

##### Comparison of Core-Shell Nanogel Degradation Products with Polymer Prepared in the Absence of Crosslinker

**Figure 7:**
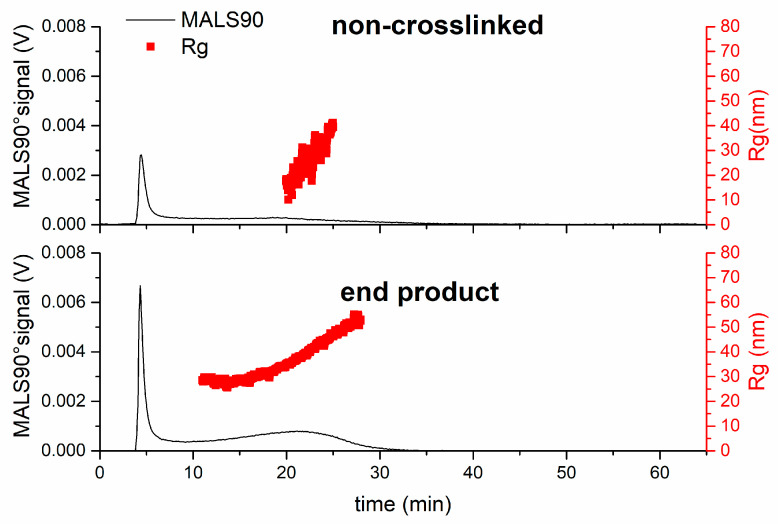
Comparison of the fractograms of the product of degradation of the core-shell nanogel after 27 h to a non-crosslinked polymerisation of NIPMAM, and then NIPAM, after synthesis.

In order to investigate the extent of crosslink cleavage, the final degradation products (after 27 h) were compared to the products of NIPMAM and NIPAM polymerisation in the absence of crosslinker (referred to as non-crosslinked). The R_g_ values of the end-products of degradation were compared with R_g_ values of the non-crosslinked polymers (see Figure 7). After degradation, the core-shell nanogel sample was larger than the non-crosslinked sample (mode R_g_ values of 30 nm and 23 nm, respectively), as also observed for the single composition nanogel. The non-crosslinked sample also showed a slightly lower intensity MALS90° than the degraded core-shell nanogel sample, potentially indicating that a greater amount of degraded nanogel sample is above the 10 kDa cut-off of the AF4 membrane. However, the core-shell nanogel sample showed a dramatic reduction in the MALS90° signal during the degradation process, indicative of the near-complete degradation of the nanogels. The data show that, in the case of the samples tested, there was limited evidence of self-crosslinking due to the inclusion of NIPAM in the polymerisation.

### 3.3. Mechanistic Insights into Nanogel Degradation Behaviour

**Figure 8:**
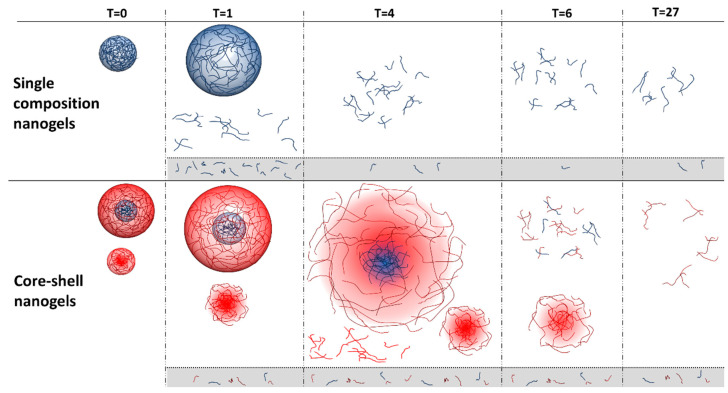
Scheme represents degradation of nanogels in the presence of DTT; red chains represents PNIPMAM polymer chains and blue chains represents PNIPAM polymer chains. For reasons of clarity, the crosslinks are not specifically shown.

By comparing the data for both the single composition nanogel and core-shell nanogel samples, it is possible to see the two different degradation behaviours provided by the samples (Figure 8). The single composition nanogel initially swells, then begins to break up into polymer fragments. These fragments are then slowly degraded to give smaller fragments over the remaining duration of analysis. It is likely that after only 1 h of degradation, much of the sample had degraded to polymer fragments smaller than 10 kDa. The core-shell nanogel was a bimodal sample and the two populations of nanogels might contain different polymer compositions. For this sample, both populations displayed swelling, then the larger population disintegrated into smaller polymer fragments much more slowly than for the single composition nanogel (Figure 8). From the presented data, it is uncertain whether the PNIPMAM core in the particles degraded at the same rate as the PNIPAM shell. The smaller population of nanogels in the core-shell sample showed even slower degradation. It is hypothesised that the slower degradation of the smaller population of nanogels in this sample may be due to them being primarily composed of PNIPAM. Future work will be needed to fully elucidate this behaviour.

## 4. Conclusions

This work addresses some of the challenges associated with characterising the degradation of nanogels. It is important to be able to measure the nanogel degradation products, as they are potential mediators of the downstream biological behaviour of these nanoparticles. By using AF4-MALS-DLS to measure the degradation of nanogels accurately, it has been possible to provide a high-resolution characterisation of the resulting polydisperse colloidal materials in a real-time manner. As the nanogels degraded, it was possible to determine the stages of the nanogel degradation, nanogel swelling, nanogel fragmentation, and then the further degradation of the polymer fragments. The different compositions of the nanogels had a significant impact on the degradation; the core-shell nanogels showed slower degradation, which might have been due to a higher degree of self-crosslinking reactions occurring in the PNIPAM shell of the nanogels. In the case of both of the nanogel samples, much of the material produced during the degradation was able to pass through the membrane of the AF4 and, therefore, can be assumed to have a molecular weight less than 10 kDa. It is likely that any polymer fragments below the molecular weight cut-off of the membrane would be cleared through the kidneys; previous reports have shown that branched polymers below 50 g mol^−1^ can undergo kidney clearance [47]. In future, it would be useful to investigate whether the degradation of the nanogels changes under more physiologically relevant conditions by using glutathione instead of DTT.

Overall, this work contributes to the growing field of nanoscience and nanomedicine by providing new insights into the design and characterisation of degradable nanogels for biomedical applications, something that can be harnessed for future product development activities.

## Figures and Tables

**Table 1:** The masses and moles of reagents used for synthesis of the four nanogel samples that were prepared.

Sample	Monomer		BAC		KPS		SDS		Water (g)	
	NIPMAM	NIPAM	Core	Shell	Core	Shell	Core	Shell	Core	Shell
Single composition PNIPMAM nanogels	4.414 g, 34.7 mmol	-	451.8 mg, 1.735 mmol	-	187.6 mg, 0.694 mmol	-	80 mg, 0.277 mmol	-	140	-
Core-shell nanogels (PNIPAM shell and PNIPMAM core)	0.6624 g, 5.250 mmol	3.340 g, 29.495 mmol	67.77 mg, 0.260 mmol	384.0 mg, 1.475 mmol	28.14, 0.104 mmol	159.46, 0.590 mmol	12 mg, 0.042 mmol	68 mg, 0.236 mmol	21	119
Single composition recipe but without crosslinker	4.414 g, 34.7 mmol	-	-	-	187.6 mg, 0.694 mmol	-	80 mg, 0.277 mmol	-	140	-
Core-shell recipe but without crosslinker	0.6624 g, 5.250 mmol	3.340 g, 29.495 mmol	-	-	28.14, 0.104 mmol	159.46, 0.590 mmol	12 mg, 0.042 mmol	68 mg, 0.236 mmol	21	119

**Table 2:** Mode values of R_g_, R_h_, and R_g_/R_h_ ratio for core-shell nanogels and the single composition nanogels samples obtained from AF4-MALS-DLS measurements.

Sample	R_g_ (nm)	R_h_ (nm)	R_g_/R_h_
Core-shell nanogels	27.4 ± 0.7 and 61.7 ± 1.5	44.0 ± 1.6 and 86.5 ± 3.2	0.61 ± 0.01 and 0.71 ± 0.02
Single composition nanogels	43.6 ± 1.2	60.4 ± 2.6	0.72 ± 0.01

## Data Availability

The data presented in this study are available on request from the corresponding author.

## Supplementary Materials

The following supporting information can be downloaded at: https://www.mdpi.com/article/10.3390/jfb14070346/s1, The supporting information contains additional characterisation of the nanogels by DLS (Figure S1 and Table S1)) and AF4 (Figures S2–S5 and Table S1). Figure S1: Particle size distribution by intensity obtained from DLS measurements for A) the core-shell nanogels and B) the single composition nanogels. Measured at 0.2% NovaChem at 28 °C; Figure S2: Reproducibility obtained from MALS90 signal for samples A) the core-shell nanogels and B) the single composition nanogels; Figure S3: AF4-MALS degradation analysis of the single composition nanogels. Fractograms showing the light 90° scattering detector signal (black, solid lines), Rg (red dotted lines) at different durations of degradation obtained from AF4-MALS-DLS measurements; Figure S4: AF4-MALS degradation analysis of the core-shell nanogels. Fractograms showing the light 90° scattering detector signal (black, solid lines), Rg (red dotted lines) at different durations of degradation obtained from AF4-MALS-DLS measurements; Figure S5: Changes in the Rg/Rh values for the core-shell nanogels in the first 2 h of degradation. Dotted lines show the position of the mode of the two populations at t = 0 h to help visualise the shift in elution time; Table S1: Comparison of the radii of the two nanogel samples the core-shell nanogels or the single composition nanogels as obtained by different characterisation methods.
