# Supplementary material for: Understanding the Degradation of Core-Shell Nanogels Using Asymmetrical Flow Field Flow Fractionation
Source: J Funct Biomater. 2023 Jun 29;14(7):346. doi: 10.3390/jfb14070346 (PMC10381601; doi:10.3390/jfb14070346)
Supplement: Supplementary file 1 [file jfb-14-00346-s001.zip › jfb-2445069-supplementary.pdf]

Supporting Information

Understanding the degradation core-shell nanogels using asymmetrical flow field flow fractionation

Edyta Niezabitowska, Dominic Gray, Eduardo Gallardo-Toledo, Andrew Owen, Steve P. Rannard and Tom O. McDonald

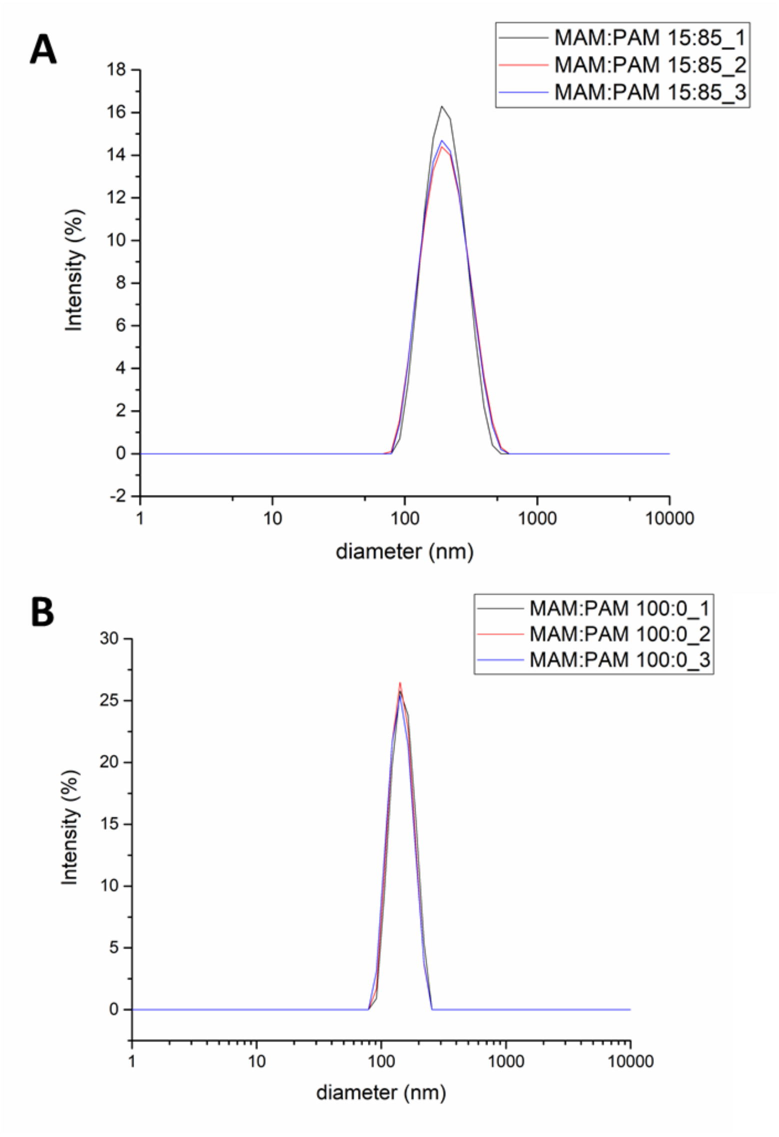

Fig S1. Particle size distribution by intensity obtained from DLS measurements for A) the core-shell nanogels and B) the single composition nanogels. Measured at 0.2% NovaChem at 28 °C.

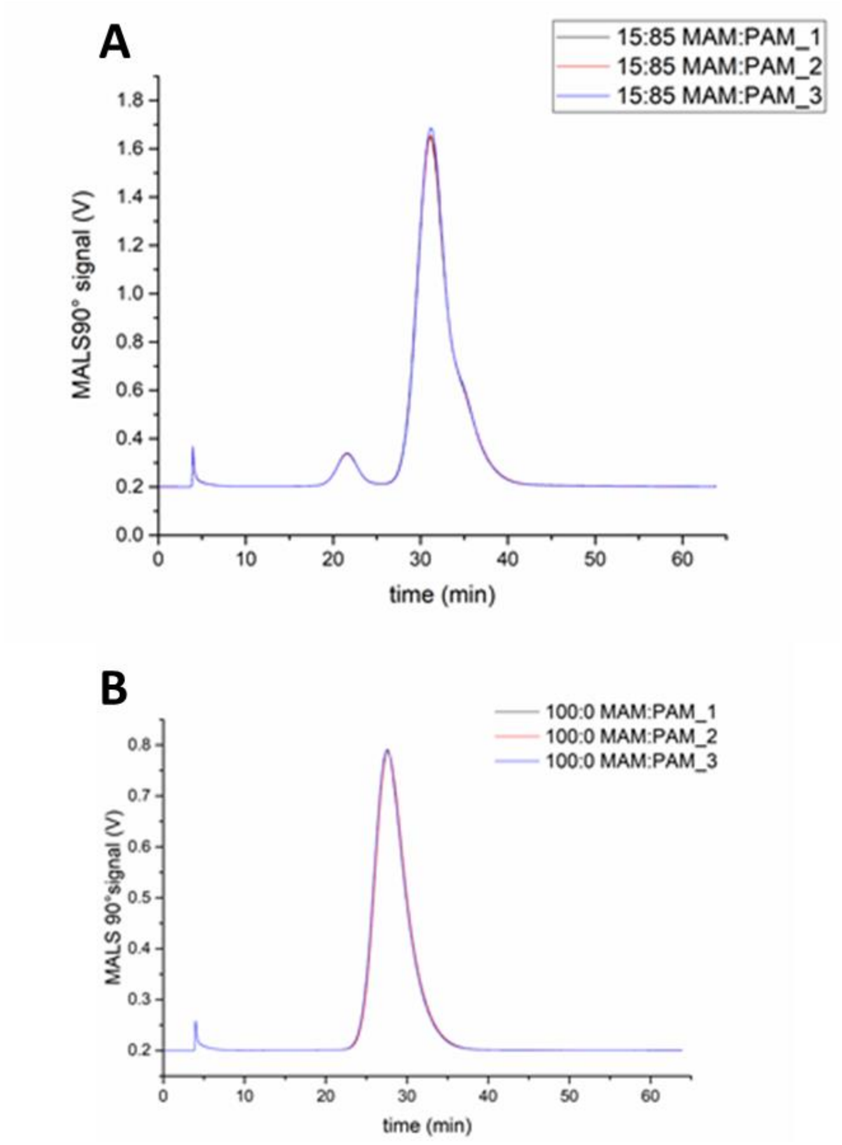

Fig S2. Reproducibility obtained from MALS90 signal for samples A) the core-shell nanogels and B) the single composition nanogels.

Table S1. Comparison of the radii of the two nanogel samples the core-shell nanogels or the single composition nanogels as obtained by different characterisation methods.

Sample	SEM (nm)	Batch DLS R_h (nm)	AF4	
			R_g (nm)	R_h (nm)
Core-shell nanogels	~<10 and 40	94 ± 1	27.4 ± 0.7 and 61.7 ± 1.5	44.0 ± 1.6 and 86.5 ± 3.2
Single composition nanogels	58	72 ± 2	43.6 ± 1.2	60.4 ± 2.6

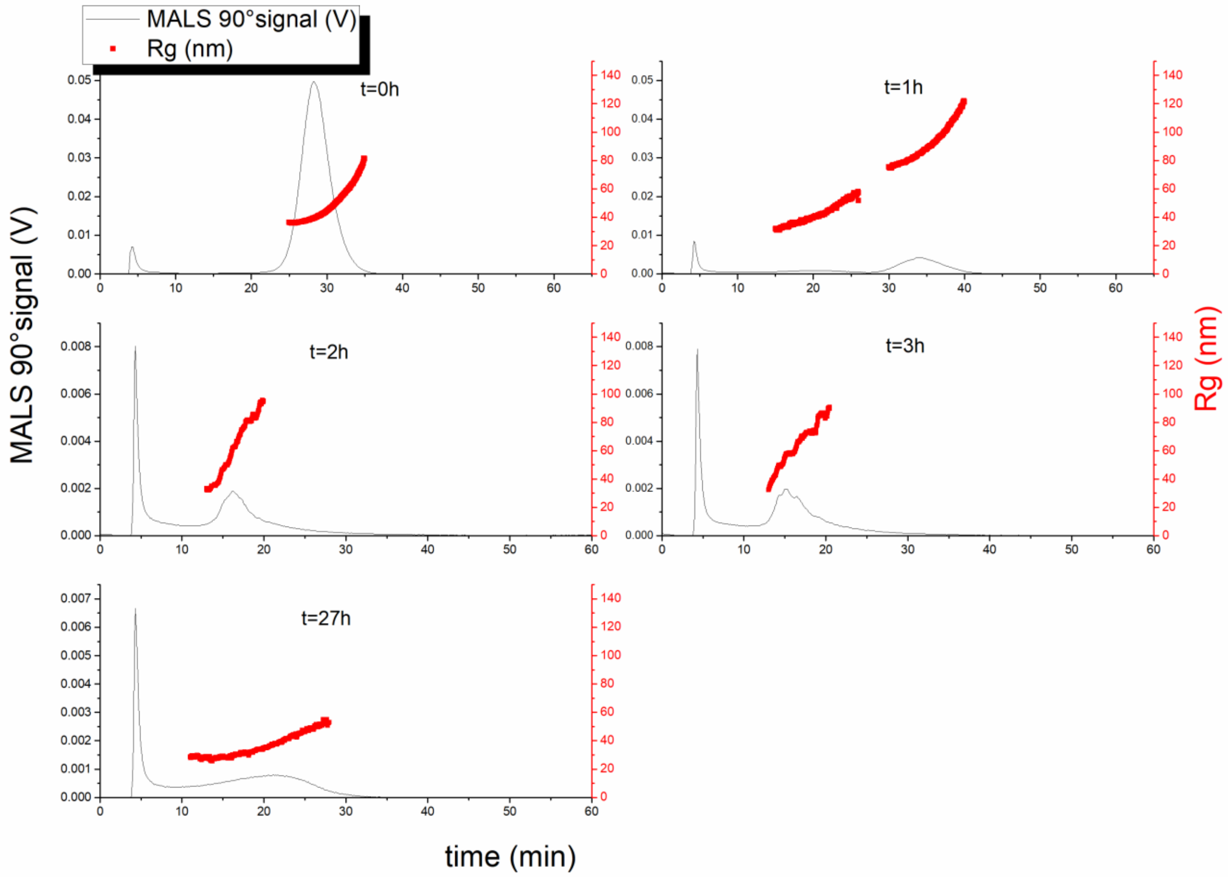

Fig. S3. AF4-MALS degradation analysis of the single composition nanogels. Fractograms showing the light 90° scattering detector signal (black, solid lines), Rg (red dotted lines) at different durations of degradation obtained from AF4-MALS-DLS measurements.

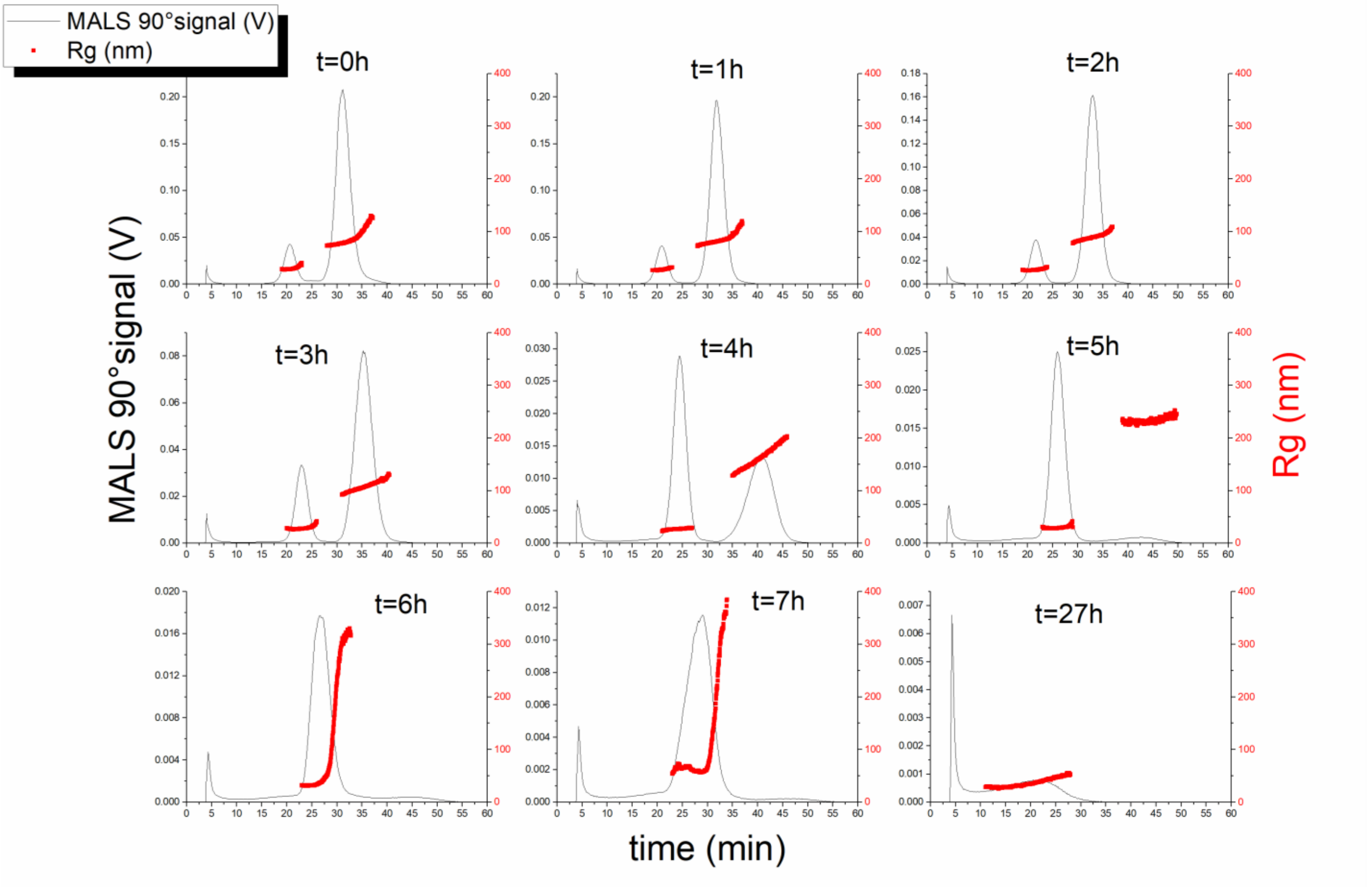

Fig. S4. AF4-MALS degradation analysis of the core-shell nanogels. Fractograms showing the light 90° scattering detector signal (black, solid lines), Rg (red dotted lines) at different durations of degradation obtained from AF4-MALS-DLS measurements.

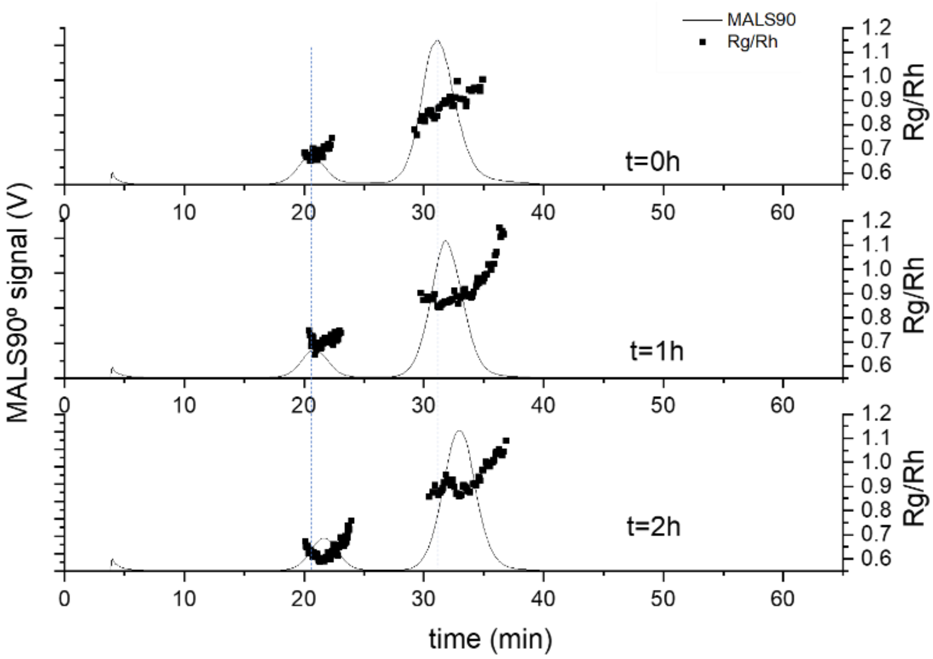

Figure S5. Changes in the R_g/R_h values for the core-shell nanogels in the first 2 hours of degradation. Dotted lines show the position of the mode of the two populations at t=0h to help visualise the shift in elution time.
